# Cauchy hyper-graph Laplacian nonnegative matrix factorization for single-cell RNA-sequencing data analysis

**DOI:** 10.1186/s12859-024-05797-4

**Published:** 2024-04-29

**Authors:** Gao-Fei Wang, Longying Shen

**Affiliations:** School of Computer Science, Qufu Normal University, Rizhao, 276826 Shandong China

**Keywords:** Single-cell RNA sequencing, Cauchy loss function, Hyper-graph regularization, Sample clustering, Non-negative matrix factorization

## Abstract

Many important biological facts have been found as single-cell RNA sequencing (scRNA-seq) technology has advanced. With the use of this technology, it is now possible to investigate the connections among individual cells, genes, and illnesses. For the analysis of single-cell data, clustering is frequently used. Nevertheless, biological data usually contain a large amount of noise data, and traditional clustering methods are sensitive to noise. However, acquiring higher-order spatial information from the data alone is insufficient. As a result, getting trustworthy clustering findings is challenging. We propose the Cauchy hyper-graph Laplacian non-negative matrix factorization (CHLNMF) as a unique approach to address these issues. In CHLNMF, we replace the measurement based on Euclidean distance in the conventional non-negative matrix factorization (NMF), which can lessen the influence of noise, with the Cauchy loss function (CLF). The model also incorporates the hyper-graph constraint, which takes into account the high-order link among the samples. The CHLNMF model's best solution is then discovered using a half-quadratic optimization approach. Finally, using seven scRNA-seq datasets, we contrast the CHLNMF technique with the other nine top methods. The validity of our technique was established by analysis of the experimental outcomes.

## Introduction

Studying a single cell reveals complex biochemical processes [1, 2]. Processing ScRNA-seq data presents distinct computational challenges as it involves the high dimensionality of the data, the existence of disruptions, and technological quirks [3, 4]. Negative matrix factorization (NMF) is a commonly used technique to reduce dimensionality and extract features from single-cell RNA sequencing (scRNA-seq) data [5, 6]. The conventional non-negative matrix factorization (NMF) techniques may not be able to accurately represent the intrinsic structure and interconnections present in the data [7, 8]. The combination of Cauchy Hypergraph Laplacian Non-Negative Matrix Factorization (CHL-NMF) uses hyper-graph Laplacian regularization in conjunction with Cauchy distribution-based sparsity to improve the robustness and interpretability of scRNA-seq data analysis [9]. With the advancement of single-cell RNA sequencing (scRNA-seq) technology in recent years, a vast amount of scRNA-seq data has been generated [10]. Researchers [11], delve into the wealth of biological insights inherent in scRNA-seq data by scrutinizing cell information and uncovering heterogeneity among cells, thereby offering valuable insights into the relationships between cells, genes, and diseases [12].

Clustering is a common method to analyze gene expression data [13]. Traditional clustering techniques include K-means and spectral clustering (SC), among others [14, 15]. The efficacy of conventional clustering approaches is significantly impacted by the high dimension, high noise, and high sparsity of single-cell RNA-seq data. Numerous innovative single-cell clustering techniques have thus far been put forth by researchers [16, 17]. As an illustration, Lu, Wang, Liu, Zheng and Kong [18] introduced SinNLRR, an enhanced Low-rank Representation (LRR) approach that adds non-negative restrictions to the LRR model. To determine how closely related cells are, this approach can map the data into the many subspaces to which it is assumed that the data belong. A multi-kernel learning approach dubbed SIMLR was put out by Guo, Wang, Hong, Li, Yang and Du [19]. The key concept of this approach is the adaptive selection of several kernel functions to measure the various data sets, ensuring that it is broadly applicable. To combine several basic partitions into consistent partitions that are as consistent as feasible with the basic partitions, Liu, Zhao, Fang, Cheng, Fu and Liu [20], introduced a technique known as entropy-based consensus clustering (ECC). Additionally, the high noise and high dimension in high-throughput sequencing data may be successfully addressed by this strategy. Using variance analysis, Bhattacharjee and Mitra [21] created the Corr clustering technique. This algorithm's benefit is that it can quickly ascertain how many clusters there are, which helps it recognize cell types more accurately.

In the context of higher-order spatial structure in the original data, the aforementioned strategies are unable to lessen the influence of noise. The large dimension makes the dimensionality reduction of the data before clustering a typical practice [22]. As a reliable approach for reducing the dimensionality of data, non-negative matrix factorization (NMF) is frequently employed in data analysis activities [23]. NMF is a traditional dimension reduction technique that has been used in a wide variety of applications [24–26].

We created the Cauchy Hyper-graph Laplacian Non-negative Matrix Factorization technique (CHLNMF) for single-cell data clustering to overcome the issues raised above. To lessen the effect of noise, CHLNMF specifically substitutes the Euclidean distance in the conventional NMF with the Cauchy loss function (CLF). To maintain the higher-order manifold structure found in the original data, the hyper-graph regularisation term is also included in the model. The deconstructed coefficient matrix is then clustered using the K-means method as per the investigations of Liu, Cao, Gao, Yu and Liang [27].

This Study suggests a fresh approach for processing and analyzing single-cell datasets, named CHLNMF. In this model, we replace the Euclidean distance used in the original NMF model with CLF, which reduces the impact of noise and improves the stability of the model. Second, the CHLNMF techniques include regularisation terms for hyper-graphs to maintain the original data's manifold structure. The non-convex optimization issue is changed into an iterative weighted problem using the half-quadratic (HQ) optimization approach, and the efficient iterative updating rules of the proposed model are derived. To test the viability of the CHLNMF approach, we ran many studies on scRNA-seq data sets. Experimental findings demonstrate that our strategy outperforms other methods in terms of overall performance.

## Materials and methods

### Non-negative matrix factorization

High-dimensional data may be handled with NMF [15], which denotes the number of genes and samples, respectively, in a non-negative matrix of dimensions. The goal of NMF is to identify two non-negative matrices that meet two requirements [16]. It must be much smaller than and is the first requirement. The second requirement is that the product of these two matrices comes close to matching the matrix. The following describes NMF's objective function:

1 $$\min \parallel {\mathbf{X}} - {\mathbf{UV}}\parallel_{F}^{2} ,s.t.{\mathbf{U}} \ge 0,{\mathbf{V}} \ge 0,$$

where denotes the Frobenius norm. The updating rules are as below [28]:

2 $$u_{ik} \leftarrow u_{ik} \frac{{({\mathbf{XV}})_{ik} }}{{{\mathbf{(UVV}})_{ik} }},$$

3 $$v_{kj} \leftarrow v_{kj} \frac{{({\mathbf{U}}^{T} {\mathbf{X}})_{kj} }}{{({\mathbf{U}}^{T} {\mathbf{UV}})_{kj} }}.$$

### Cauchy loss function

In nature, noise is prevalent in data processing. Meanwhile, they are, for the most part, complex and unknown. Therefore, how effectively overcoming the impact of noise is crucial when analyzing data. The CLF is a reliable loss function that has been used for face recognition and picture clustering. In addition to improving the model’s resilience to non-Gaussian noise and outliers, CLF may effectively slow the rise of noise and outliers. According to [17], the Cauchy loss function is as follows:

4 $$f(x) = \ln \left( {1 + \frac{{x^{2} }}{{c^{2} }}} \right),$$

where the parameter controls the size of the Cauchy loss function's upward opening. In other words, when it is larger, the faster the slope of the function tends to be zero.

It is easy to see that the CLF is a natural logarithm based on the quadratic function. Due to the nature of the logarithmic function, as the independent variable increases, the slope of the function at that point will get closer and closer to zero. Therefore, when the independent variable becomes large, the Cauchy function can slow down the growth rate of the function value at this point, which can mitigate the impact of noise.

**Fig. 1:**
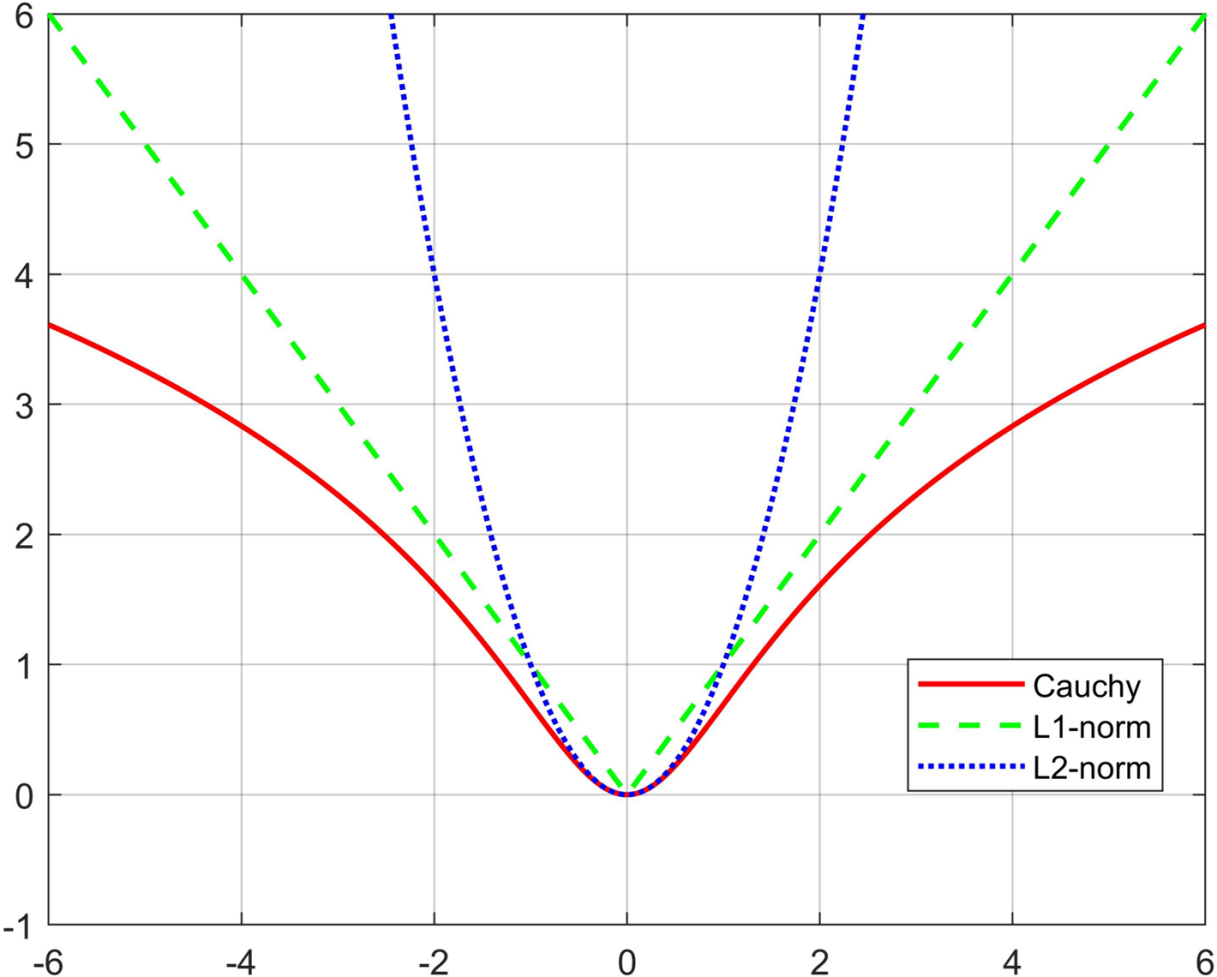
Image representation of three different loss functions

The graph of the CLF is explored in Fig. 1 and shows independent variable variability, the function value of $L_{2}$- the norm tends to infinity. When the independent variable exists at a certain point, even if there is a tiny fluctuation, the function value may change considerably. Compared with the Cauchy function, the growth of function value is restrained. Therefore, using CLF to replace the measurement based on Euclidean distance in the standard NMF model is helpful to increase the stability of the method.

### Hyper-graph regularization

There are some similarities and differences between hyper-graphs and simple graphs [29, 30]. The fact that the edges of the hyper-graph can be linked to additional nodes differs from the fact that they all take into account the original data's complex structure. As a result, the original data's higher-order spatial structure can be preserved via hyper-graph constraint.

**Fig. 2:**
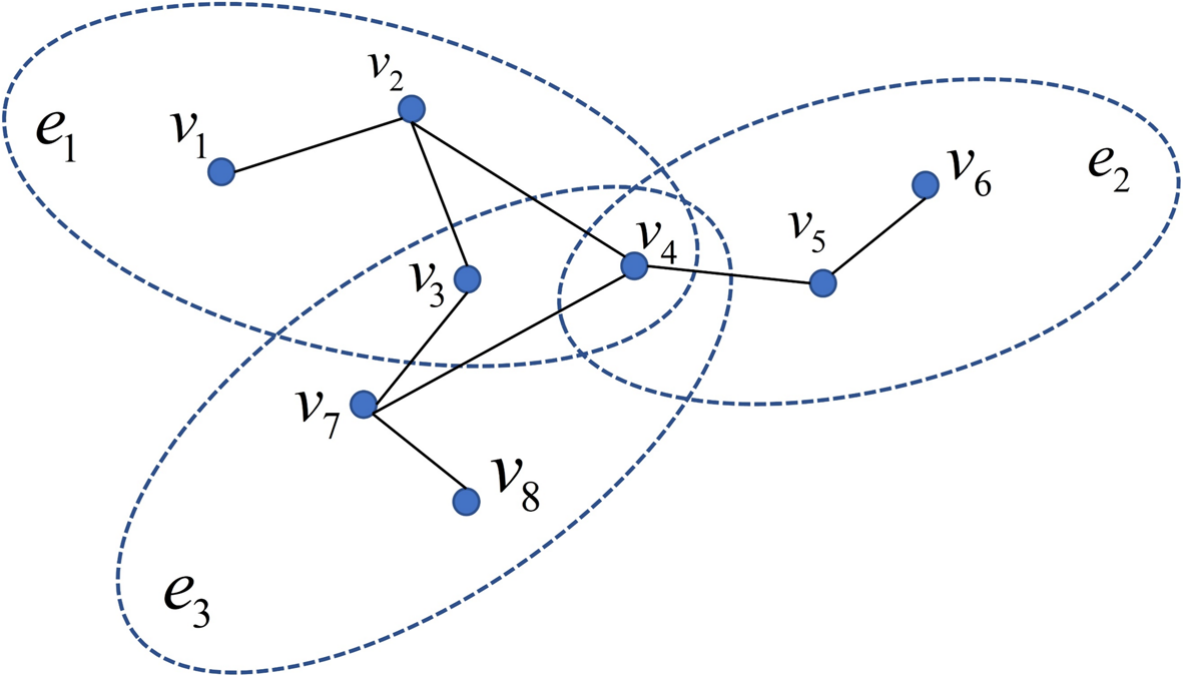
The schematic diagram of the hyper-graph

Non-empty vertex sets, non-empty hyper-edge sets, and a hyper-edge weight matrix make up a hyper-graph. Typically, a hyper-graph is expressed by ${\mathbf{G = (V,E,W)}}$, where ${\mathbf{E}} = \{ e_{i} |i = 1,2,...,n\}$ are non-empty hyper-edges sets, ${\mathbf{V}} = \{ v_{j} |j = 1,2,...,m\}$ non-empty vertex sets, and is a weight matrix of hyper-edge. $e_{i}$ is a subset of the hyper-edge set ${\mathbf{E}}$, which is a hyper-edge. Each includes a lot of vertexes $v_{j}$. Figure 2 illustrates the hyper-graph's structural layout.

In a schematic diagram of the hyper-graph, vertexes are data points and each vertex exists in one or more hyper-edges, such as belongs to hyper-edge and $e_{3}$. At the same time, each hyper-edge has multiple vertices, such as a hyper-edge $e_{2}$ containing three vertices, which are $v_{4}$, $v_{5}$ and $v_{6}$ respectively. In other words, the hyper-edge is a subset of vertex sets ${\mathbf{V}}$. Based on these basic concepts, hypergraphs have a series of related definitions.

We give each hyper-edge an initialization weight and draw a hyper-graph. Firstly, given an affinity matrix ${\mathbf{A}}$ which is defined as ${\mathbf{A}}_{{{\text{ij}}}} = \exp \left( { - {{\parallel v_{i} - v_{j} \parallel^{2} } \mathord{\left/ {\vphantom {{\parallel v_{i} - v_{j} \parallel^{2} } {\sigma^{2} }}} \right. \kern-0pt} {\sigma^{2} }}} \right),$ in which $\sigma$ represents the average separation across each vertex. The starting weight for each hyper-edge may therefore be defined as follows:

5 $${\mathbf{W}}_{i} = \sum\limits_{{V_{j} \in e_{i} }} {{\mathbf{A}}_{ij} } .$$

Usually, using the incidence matrix ${\mathbf{H}}(v,e)$ shows the relationships between a vertex and a hyper-edge. The definition ${\mathbf{H}}$ is as follows:

6 $$H(v,e) = \left\{ {\begin{array}{*{20}c} {1,} & {if} & {v \in e} \\ {0,} & {if} & {v \notin e} \\ \end{array} } \right..$$

Add the weights of all hyper-edges connected on the same vertex $v_{j} \in {\mathbf{V}}$, and the total is referred to as the vertex's degree. The degree of hyper-edge is typically the number of vertices that belong.

7 $$d(v) = \sum\limits_{{\{ e_{i} \in {\mathbf{E}}|v \in e\} }} {w(e) = } \sum\limits_{{e_{i} \in {\mathbf{E}}}} {w(e){\mathbf{H}}(v,e),}$$

8 $$\delta (e) = \left| e \right| = \sum\limits_{{v_{j} \in {\mathbf{V}}}} {{\mathbf{H}}(v,e).}$$

Given a diagonal matrix ${\mathbf{D}}_{v}$, the element ${\mathbf{D}}_{v}$ is the degree of a vertex. And define a matrix ${\mathbf{D}}_{e}$ in which elements are the degree of hyper-edge. From the literature [18], The unnormalized hyper-graph Laplacian matrix can be known. ${\mathbf{L}}_{hyper} = {\mathbf{D}}_{v} - {\mathbf{S}}$, where ${\mathbf{S}} = {\mathbf{HWD}}_{e}^{ - 1} {\mathbf{H}}^{T}$.

### Objective function of CHLNMF

NMF has been successfully used in several sectors and is an efficient dimension-reduction technique. Real-world applications typically have a lot of outliers and noise in their data. Nevertheless, non-Gaussian outliers and noise can affect the typical NMF. However, it cannot also learn the original data's high-dimensional manifold structure.

**Fig. 3:**
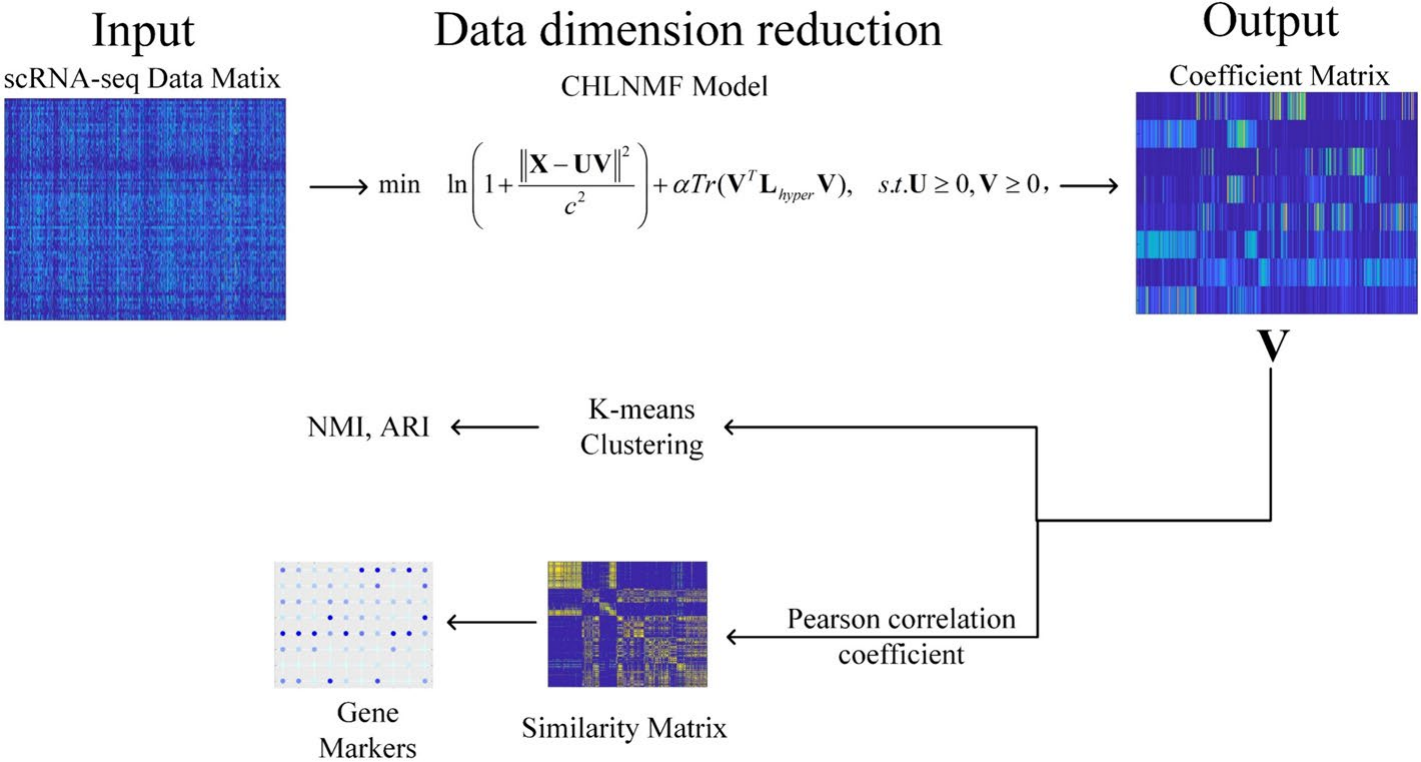
The framework of CHLNMF

An approach dubbed CHLNMF is suggested as a solution to the aforementioned problems. In particular, CLF is used instead of the traditional Euclidean distance to measure error. CLF can significantly mitigate the impact of data noise. It is beneficial to make the model more resilient. The manifold structure in high-dimensional space is preserved concurrently with the addition of the hyper-graph constraint component to the CHLNMF model. In conclusion, the objective purpose $O_{CHLNMF}$ of CHLNMF is as below:

9 $$\min \;\ln \left( {1 + \frac{{\left\| {{\mathbf{X}} - {\mathbf{UV}}} \right\|^{2} }}{{c^{2} }}} \right) + \alpha Tr({\mathbf{V}}^{T} {\mathbf{L}}_{hyper} {\mathbf{V}}),\;s.t.\;{\mathbf{U}} \ge 0,{\mathbf{V}} \ge 0,$$

where $c$ is a regularisation parameter for the hyper-graph, is the trace of the matrix, and regulates the slope's rate of descent to zero is a parameter which controls the rate of the slope going to zero, $\alpha$ is a hyper-graph regularization parameter, and $Tr( \cdot )$ is the trace of the matrix. Our model Framework is shown in Fig. 3.

### Optimization and updating rule of CHLNMF

It is challenging to directly find the optimal solution of the CHLNMF model since its objective function is non-convex. Therefore, using Semi-quadratic programming theory to solve the objective function $O_{CHLNMF}$ to find the optimal solution. The primary concept is to add an auxiliary variable and change the objective function into an enhanced objective function. According to the half-quadratic programming theory [31], The following issue is identical to the objective function in Eq. (9):

10 $$\min \left\{ {\tfrac{1}{2}\omega_{j} \left\| {{\mathbf{X}} - {\mathbf{UV}}} \right\|^{2} + \theta (\omega_{j} )} \right\} + \alpha Tr({\mathbf{V}}^{T} {\mathbf{L}}_{hyper} {\mathbf{V}}),\;s.t.\;\omega ,{\mathbf{U}},{\mathbf{V}} \ge 0,$$

where $\theta (\omega_{j} )$ is a conjugate of Cauchy functions and $\omega_{j}$ is an auxiliary variable. Three variables need to be optimized in this optimization problem; therefore, it can be solved by alternating iteration updates.

1. Fixed $\omega$, solve for ${\mathbf{U}}$ and ${\mathbf{V}}$:

Because of the fixed $\omega$, The following issue is produced by reducing Eq. (10):

11 $$\min \tfrac{1}{2}\omega_{j} \left\| {{\mathbf{X - UV}}} \right\|^{2} + \alpha Tr({\mathbf{V}}^{T} {\mathbf{L}}_{hyper} {\mathbf{V}}),s.t.{\mathbf{U}},{\mathbf{V}} \ge 0.$$

To solve this problem, ${\mathbf{U}} \ge 0$ and ${\mathbf{V}} \ge 0$ are constrained through two introduced Lagrange multipliers ${{\varvec{\uppsi}}} = \left[ {\psi_{ik} } \right]$ and ${\mathbf{\varphi }} = \left[ {\varphi_{kj} } \right]$, respectively. And then, we obtain a Lagrange function. Which show as follows:

12 $$L = Tr({\mathbf{\Lambda XX}}^{T} ) - 2Tr({\mathbf{\Lambda XV}}^{T} {\mathbf{U}}^{T} ) + Tr({\mathbf{\Lambda UVV}}^{T} {\mathbf{U}}^{T} ) + \alpha Tr({\mathbf{V}}^{T} {\mathbf{LV}}) + Tr({\mathbf{\psi U}}^{T} ) + Tr{\mathbf{(\varphi V}}^{T} ),$$

where $$\Lambda = diag(\omega ).$$

The partial derivative of the function $L$ is obtained concerning ${\mathbf{U}}$ and ${\mathbf{V}}$, respectively:

13 $$\frac{\partial L}{{\partial {\mathbf{U}}}} = - 2{\mathbf{\Lambda XV}}^{T} + 2{\mathbf{\Lambda UVV}}^{T} + {{\varvec{\uppsi}}},$$

14 $$\frac{\partial L}{{\partial {\mathbf{V}}}} = - 2{\mathbf{U}}^{T} {\mathbf{\Lambda X}} + 2{\mathbf{U\Lambda UV}}^{T} + 2\alpha {\mathbf{LV}} + {\mathbf{\varphi }}.$$

According to the Karush-Kuhn-Tucher (KKT) conditions, let ${\mathbf{\psi U}} = 0$ and ${\mathbf{\varphi V}} = 0$. Updating rules are as below [32]:

15 $$u_{ik} = u_{ik} \frac{{({\mathbf{\Lambda XV}}^{T} )_{ik} }}{{({\mathbf{\Lambda UVV}}^{T} )_{ik} }},$$

16 $$v_{kj} = v_{kj} \frac{{({\mathbf{U}}^{T} {\mathbf{\Lambda X}} + \alpha {\mathbf{SV}})_{kj} }}{{({\mathbf{U}}^{T} {\mathbf{\Lambda UV}} + \alpha {\mathbf{D}}_{v} {\mathbf{V}})_{kj} }}.$$

(2) Fixed ${\mathbf{U}}$ and ${\mathbf{V}}$, solve for $\omega$:

Because of the fixed ${\mathbf{U}}$ and ${\mathbf{V}}$, the Eq. (11) is reduced to the following problem:

17 $$\min \;\{ \tfrac{1}{2}\omega_{j} \left\| {{\mathbf{X - UV}}} \right\|^{2} + \theta (\omega_{j} )\} ,\;s.t.\omega \ge 0.$$

The best answer to this issue is clear, and it looks like this:

18 $$\omega_{j}^{*} = \frac{2}{{c^{2} + \left\| {{\mathbf{X}} - {\mathbf{UV}}} \right\|^{2} }}.$$

In conclusion, the detailed process of the CHLNMF algorithm is shown in Algorithm 1:

**Algorithm 1:**
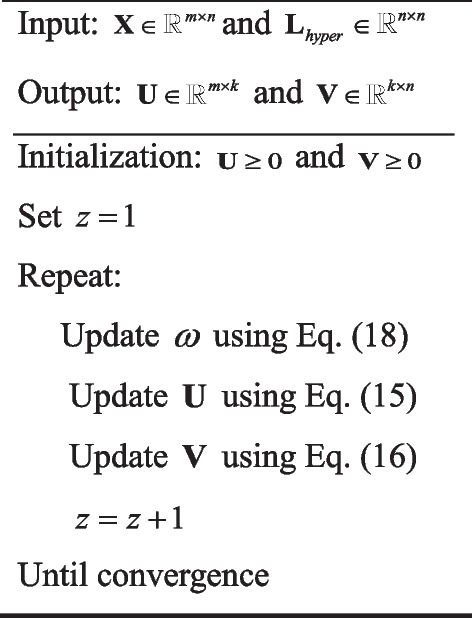
CHLNMF

### Data sets

The data sets can download from the NCBI (http://www.ncbi.nlm.nih.gov/) and EMBL-EBI (http://www.ebi.ac.uk/arrayexpress/), including Pollen [33], Grover [34], Deng [35], Darmains [36], Goolam [37], Treutlin [38], and Ting [39]. The details of seven scRNA-seq data sets were summarized in Table 1.

**Table 1:** Detailed information of seven datasets

Datasets	Cells	Genes	Cell types
Pollen	249	14805	11
Grover	135	14739	2
Deng	135	12548	7
Darmanis	420	22085	8
Goolam	124	40315	5
Treutlein	80	959	5
Ting	114	14405	5

### Evaluation metrics

In the experiment, we utilise NMI and ARI as evaluation indexes of experimental performance. The NMI is defined as:

19 $$NMI(Q,J) = \frac{M(Q,J)}{{[IE(Q) + IE(J)]/2}},$$

where $IE( \cdot )$ and $M( \cdot , \cdot )$ reflect the mutual information and the entropy of the information, accordingly. $Q = \{ Q_{1} ,Q_{2} ,...,Q_{k} \}$ and $J = \{ J_{1} ,J_{2} ,...,J_{k} \}$ represent the actual cell clusters and the anticipated labels, accordingly.

The ARI is defined as:

20 $$RI(Q,J) = \frac{{\sum\nolimits_{{{\text{ij}}}} {\left( {\begin{array}{*{20}c} {d_{ij} } \\ 2 \\ \end{array} } \right) - \left[ {\sum\nolimits_{ij} {\left( {\begin{array}{*{20}c} {d_{ij} } \\ 2 \\ \end{array} } \right)} \sum\nolimits_{ij} {\left( {\begin{array}{*{20}c} {d_{ij} } \\ 2 \\ \end{array} } \right)} /\left( {\frac{d(d - 1)}{2}} \right)} \right]} }}{{\tfrac{1}{2}\left[ {\sum\nolimits_{i} {\left( {\begin{array}{*{20}c} {o_{i} } \\ 2 \\ \end{array} } \right)} + \sum\nolimits_{j} {\left( {\begin{array}{*{20}c} {k_{j} } \\ 2 \\ \end{array} } \right)} } \right] - \left[ {\sum\nolimits_{i} {\left( {\begin{array}{*{20}c} {o_{i} } \\ 2 \\ \end{array} } \right)} + \sum\nolimits_{j} {\left( {\begin{array}{*{20}c} {k_{j} } \\ 2 \\ \end{array} } \right)} } \right]/\left( {\frac{d(d - 1)}{2}} \right)}},$$

where $d_{ij}$ represents the mean of $Q_{i}$ and $J_{j}$. $o_{i}$ and $k_{i}$ shows how many cells are in the cluster. $Q_{i}$ and $J_{j}$, correspondingly.

### Model convergence analysis

**Fig. 4:**
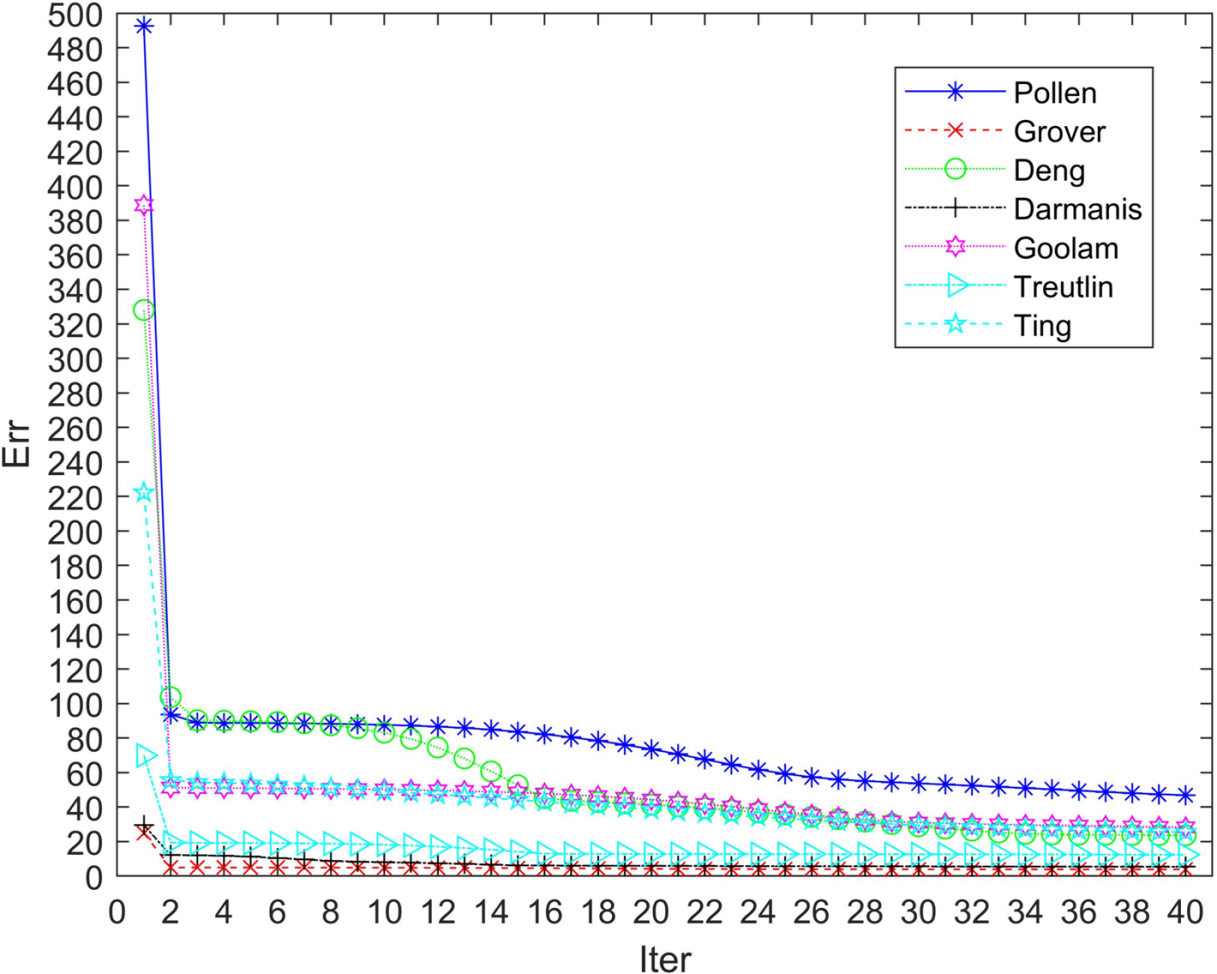
Convergence curves of CHLNMF on seven data sets

To ensure comparability in our numerical studies, we standardized all algorithms by implementing a learning rate of 0.01 and a convergence threshold of 100 iterations. In addition, we used a random bootstrap method and applied a dimensionality reduction method, such as PCA, before clustering. This was done to ensure a fair comparison of algorithms for all participants. The CHLNMF model used Stochastic Gradient Descent (SGD) with a learning rate of 0.001 for optimization. It incorporated hyperparameters such as five clusters or hypergraphs, regularization parameters λ1 = 0.1 and λ2 = 0.01, and parameters α = 0.5 and β = 0.1 for the Cauchy loss function. The goal of developing the CHLNMF model for processing single-cell RNA sequencing data was to provide accurate clustering results and efficient dimensionality reduction. The selection of these properties was based on preliminary tests and theoretical considerations. We verified the convergence of the CHLNMF model through experiments, as shown in Fig. 4, representing the error value converges to a certain range within five iterations, proving that our algorithm converges rapidly.

## Results and discussion

### Parameters setting

In the CHLNMF model, two parameters need to be determined: the hyper-graph regularization parameter $\alpha$, and the scale factor of the CLF $c$. Verifying the impact of parameters on the model requires: Our team have carried out corresponding experiments, and the experimental results are as follows.

**Fig. 5:**
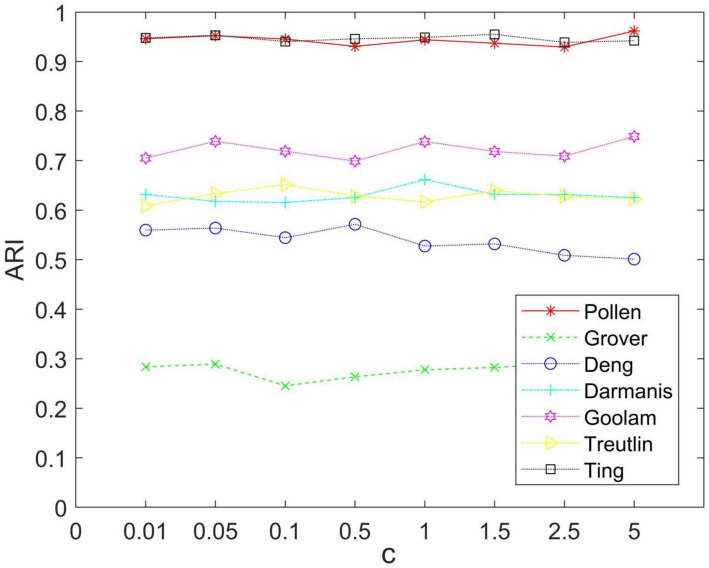
Performance of CHLNMF on seven datasets when $c$ taking different values

For the scale parameter $c$, we take eight values in the range of 0.01 to 5 to verify its impact on seven scRNA-seq data sets and selected ARI as the evaluation index. In Fig. 5, the experimental findings are displayed. The model's illustrative figure makes this clear, strong robustness to the parameter $c$, and the model is less dependent on it $c$. Therefore, the parameter is set to 0.5 in subsequent experiments.

**Fig. 6:**
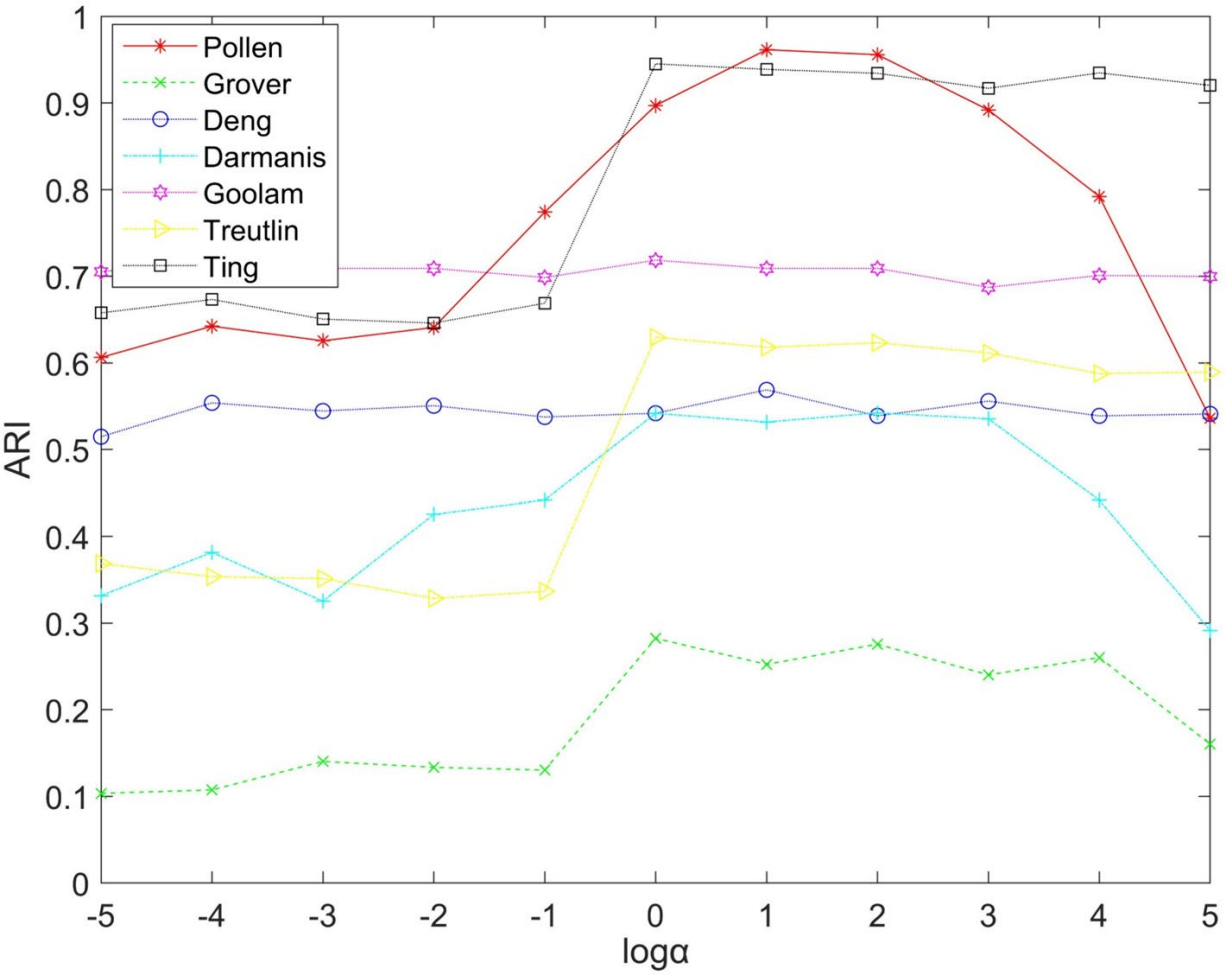
Performance of CHLNMF on seven datasets when $\alpha$ taking different values

For the hyper-graph regularization parameter $\alpha$, its size affects the learning degree of higher-order space structure. In the experiment, $\alpha$ is set in $\{ 10^{t} \left| {r \in [ - 5, - 4, - 3,...,3,4} \right.,5]\}$. The outcomes of the experiment are displayed in Fig. 6. The parameter significantly affects the model's performance in the majority of data sets. When the parameter is set to $10^{1}$, the model performs better in all data sets. Therefore, the parameter is set to in subsequent experiments.

### Clustering results analysis

To demonstrate the efficacy of the CHLNMF approach, we ran it on seven human or mouse scRNA-seq datasets. Aside from that, we used SinNLRR [40], ECC [20], Corr [41], SIMLR [42], SC [43], SSC [44], K-means [45], PCA [46], and t-SNE [47] as comparison methods. The matrix were obtained after the dimensionality reduction of the original data by the CHLNMF model, and K-means clustering is performed on the coefficient matrix ${\mathbf{V}}$. Except for the Corr method, the number of the cell population of methods is known in the clustering process. NMI values range from 0 to 1, while the values of ARI are between − 1 and 1. Performance improves as the index value increases. Tables 2, 3, and 4 display the clustering results, and we may infer the following findings:

1. The CHLNMF technique is an enhanced iteration of the NMF approach that can efficiently reduce the dimension of single-cell RNA sequencing data, identify various cell types using the coefficient matrix produced after processing, and discover cell heterogeneity. The experimental findings of NMI and ARI in Tables 2 and 3 demonstrate that the low-rank subspace model performs very well in classifying various cell types. However, because it takes into account the effects of noise and manifold structure in high-dimensional data, CHLNMF performs better overall than the SinNLRR technique. The PCA, CHLNMF, and SinNLRR methods all decompose the data by matrix, but the characteristic solution of principal component analysis is gained through neutralization, It's not sensitive to cell heterogeneity, so its performance is worse than CHLNMF and SinNLRR.
2. Tables 2 and 3 provide the parameters that can be used to further analyze the performance of the CHLNMF model against the K-means technique. When comparing the two models, the CHLNMF model consistently produces better normalized mutual information (NMI) values (from 0.9142 to 0.9632) compared to the K-means model (from 0.7174 to 0.8813). Furthermore, the CHLNMF model applies to all data sets. CHLNMF outperforms K-means in terms of NMI values, with an average increase of about 11% to 14%. Adjusted Rand Index (ARI) values for CHLNMF range from approximately 0.805 to 0.9501, while those for K-means range from 0.3453 to 0.8567. This difference is consistent across all datasets. When comparing K-means with CHLNMF, it is seen that CHLNMF consistently achieves ARI values that are around 15% to 30% higher. This indicates a considerable improvement in both clustering accuracy and agreement with the real labels. The results are consistent with previous research [48, 49], that has shown the limitations of using K-means and other traditional clustering techniques on high-dimensional, noisy scRNA-seq data. Previous research [50] has emphasized the importance of clustering algorithms' ability to withstand and filter out noise to represent the intrinsic biodiversity found in single-cell datasets accurately. The superior performance of the CHLNMF model indicates the effectiveness of new techniques such as hypergraph regularization and the Cauchy loss function. This is in line with the goals of previous research [51], efforts aimed at improving the precision and reliability of clustering in single-cell transcriptome analysis. Given the challenges of working with complex and noisy single-cell datasets, our driven model contributes to ongoing efforts to develop advanced computational methods for analyzing scRNA-seq data. The superior performance of the CHLNMF model demonstrates its potential as a robust method to gain meaningful insights from scRNA-seq data in many biological scenarios and solve complex problems as earlier seen in multiple studies [52, 53].
3. Different clustering methods demonstrate varying levels of performance when applied to single-cell RNA sequencing (scRNA-seq) data. The basic techniques, such as K-means, t-SNE, and SCC, provide satisfactory performance with average ARI scores ranging from approximately 0.805 to 0.9592. In contrast, these less intricate techniques exhibit higher average ARI scores compared to more complicated ones, such as SIMLR and Corr. When it comes to capturing complex data structures and relationships between cells, SIMLR and Corr perform exceptionally well, achieving average Adjusted Rand Index (ARI) scores of 0.9415 and 0.9803, respectively. Although basic clustering methods are straightforward, they still achieve competitive Adjusted Rand Index (ARI) scores, making them suitable for analyzing single-cell RNA sequencing (scRNA-seq) data. However, the better ARI scores achieved by SIMLR and Corr indicate that not all modifications to traditional methods result in improved performance. Researchers must carefully evaluate the suitability of clustering algorithms based on the distinct characteristics and goals of their scRNA-seq datasets.
4. Tables 2 and 3 show that our technique outperforms previous NMI index and ARI index methods on the Pollen, Grover, Deng, and Darmanis data sets. On the remaining three datasets, it outperforms the majority of techniques as well. Table 4 presents a summary of the performance of different clustering algorithms on seven datasets, indicating that CHLNMF performs better than the other methods. The integration of the Cauchy loss function and the preservation of the manifold structure using hypergraphs be effective in improving the understanding of cell properties. CHLNMF has the highest level of agreement between real and projected clusters, as seen by its superior average ARI and NMI values compared to the other investigated methods. A sensitivity of 0.85 and a specificity of 0.72 for CHLNMF explored that the method correctly identifies 85% of positive instances and 72% of negative instances, highlighting its strength in capturing diverse data patterns. Therefore examining the specificity and sensitivity relative to the ARI and NMI can provide a comprehensive assessment that highlights each tool's ability to reliably identify positive and negative instances. This illustrates the potential benefits of CHLNMF to effectively capture complex data structures and emphasizes the need to use many evaluation metrics to gain a better understanding of the performance of clustering methods.

**Table 2:** The result of NMI on seven data sets

NMI	Pollen	Grover	Deng	Darmanis	Goolam	Treutlin	Ting
CHLNMF	0.9632	0.2381	0.7588	0.7689	0.7821	0.7868	0.9355
SinNLRR	0.9235	0.2218	0.7289	0.7433	0.8715	0.8328	0.8805
ECC	0.8859	0.2217	0.7218	0.5491	0.4556	0.6322	0.7897
Corr	0.8799	0.1582	0.6799	0.7594	0.5729	0.6744	0.7945
SIMLR	0.9428	0.0697	0.7419	0.6055	0.5599	0.6815	0.9744
SC	0.9363	0.1717	0.6757	0.5826	0.5910	0.8196	0.9515
SSC	0.9477	0.1376	0.6559	0.5836	0.5807	0.7102	0.9645
K-means	0.9142	0.2080	0.7174	0.4654	0.5686	0.7157	0.8813
PCA	0.9234	0.2125	0.7270	0.4445	0.6253	0.7530	0.8944
t-SNE	0.9190	0.2197	0.7155	0.6021	0.7043	0.7346	0.7768

**Table 3:** The result of ARI on seven data sets

ARI	Pollen	Grover	Deng	Darmanis	Goolam	Treutlin	Ting
CHLNMF	0.9501	0.2892	0.5419	0.6185	0.7568	0.6265	0.9406
SinNLRR	0.9022	0.2831	0.4651	0.5988	0.8848	0.6358	0.8843
ECC	0.8050	0.2871	0.4918	0.3164	0.3202	0.4801	0.6238
Corr	0.7553	0.1055	0.4753	0.6183	0.3046	0.4919	0.6302
SIMLR	0.9415	0.0946	0.4565	0.3982	0.2991	0.5114	0.9803
SC	0.9013	0.2261	0.3917	0.5258	0.4445	0.6191	0.9592
SSC	0.9292	0.1849	0.3804	0.5202	0.4441	0.5242	0.9784
K-means	0.8378	0.2712	0.4914	0.3453	0.4182	0.6172	0.8567
PCA	0.8886	0.2712	0.4815	0.3278	0.4594	0.5727	0.8761
t-SNE	0.8055	0.2712	0.5301	0.5725	0.5255	0.5473	0.6384

**Table 4:** The average ARI and NMI on seven data sets

	ARI (average)	NMI (average)	Sensitivity	Specificity
CHLNMF	0.6748	0.7476	0.85	0.72
SinNLRR	0.6649	0.7432	0.78	0.81
ECC	0.4749	0.6080	0.62	0.67
Corr	0.4830	0.6456	0.73	0.58
SIMLR	0.5259	0.6537	0.68	0.74
SC	0.5811	0.6755	0.75	0.69
SSC	0.5659	0.6541	0.71	0.72
K-means	0.5482	0.6387	0.65	0.68
PCA	0.5539	0.6543	0.70	0.66
t-SNE	0.5558	0.6674	0.72	0.71

### Gene markers prioritization result analysis

**Fig. 7:**
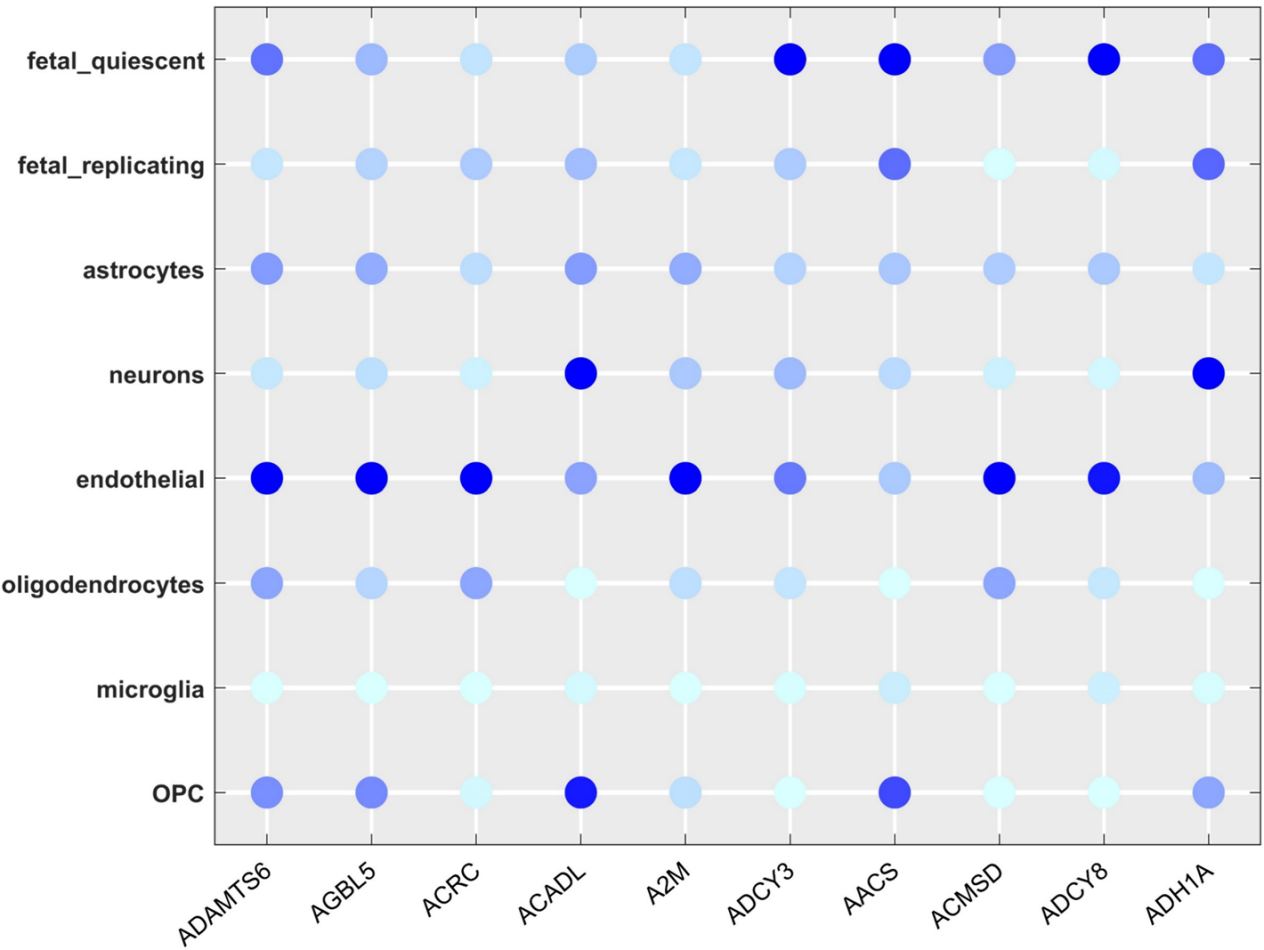
The top 10 gene markers in Darmanis data sets

The prioritization of gene markers has always been the focus of attention. There are many of unknown biological information in cell gene markers which is very helpful for us to distinguish cell subpopulations and discover the complexity of cells [54, 55]. In our experiment, firstly, the original data are processed by the CHLNMF model to attain the coefficient matrix ${\mathbf{V}}$. The similarity matrix was created using the learned similarity of the coefficient matrix and Pearson's coefficient. Following that, we utilized the Laplacian Score to choose the genes that had a differential expression on the similarity matrix. The nearest neighbor graph is built using the Laplacian Score, which also incorporates the original gene expression matrix and similarity matrix to determine each gene's score. We predict that the gene's importance is inversely correlated with the Laplacian explored score. The markers were chosen as the genes with the highest scores and the top ten marker genes were selected according to the sequence of scoring genes from high to low as depicted in Fig. 7.

GSEA analysis**,** ADAMTS6 is full of cancer-related pathways, like VEGF, which regulates angiogenesis. Vascular endothelial growth factor signaling pathway inhibition has been demonstrated to impede cardiovascular formation, preventing the development and propagation of tumors as earlier researchers investigated [12, 56]. This indicates that ADAMTS6 is closely related to endothelial cells [57]. AACS is an enzyme that uses ketones to provide cholesterol [58]. The DNA of the AACS promoter in the rat fetal adrenal is hypermethylated as a result of prenatal nicotine exposure. These modifications may lower AACS expression and cholesterol supply, which would impede the fetal adrenal gland's ability to produce steroids. Other genes are nevertheless interesting to research even though their precise roles are yet unknown. The study of these genes may be given greater focus in the subsequent effort, which will lead to the discovery of more useful data.

## Conclusions

The fast advancement of scRNA-seq technology has led to the discovery of an increasing amount of important single-cell data, which is very helpful for our understanding of single-cell but also presents several obstacles. In single-cell data, there are many noises and outliers, which pose challenging issues for our analysis procedure. In this study, we propose a novel approach to analyze single-cell data called CHLNMF by introducing the Cauchy loss function into the NMF model to replace the square loss in the fundamental model. The effect of noise may be lessened, as well as the method's robustness can be increased, by adding the Cauchy loss function. The model may retain more spatial information by including the hyper-graph, which will enhance the algorithm's performance. On seven scRNA-seq data sets, the experiment compares the CHLNMF model with nine sophisticated scRNA-seq data processing models. The experimental findings demonstrate that the CHLNMF model performs more comprehensively. Although the CHLNMF model has good performance, there are still many problems for us to study. We need to further find the loss function with better robustness to improve the performance of the model and find more valuable information. Prioritizing hyperparameters shows the impact on the performance of the CHLNMF model. It may be important to fine-tune or optimize the hyperparameters for certain datasets or research objectives. It is crucial to examine if the model can handle larger datasets or other types of data, since processing time and computer resources may provide limitations. Additionally, due to the assumption of non-negativity in the CHLNMF model, it may fail to capture complex data structures or intercellular interactions. Therefore, it is crucial to exercise caution when interpreting clustering results obtained from this model. Finally, it remains uncertain if the CHLNMF model can be applied to other biological scenarios and experimental conditions with confidence. Despite certain limitations, our work establishes a foundation for future research to enhance and broaden the capabilities of the CHLNMF model for processing scRNA-seq data.

### Future directions

In future work, in addition to solving the above problems, we will continue to study new single-cell analysis methods. Interpreting a large amount of information in scRNA-seq data is the direction and driving force of our future work. The important conclusions and consequences of the work should be succinctly explained in the Conclusions section, underscoring the value and significance of the work.

## Data Availability

The accession numbers of datasets Pollen, Grove, Deng, Darmanis, Goolam, Treutlein, and Ting are SRP041736, GSE70657, GSE45719, GSE67835, E-MTAB-3321, GSE52583, and GSE51372, to which the National Centre for Biotechnology Information has received submissions (NCBI) (http://www.ncbi.nlm.nih.gov/). And accession number of dataset Goolam is E-MTAB-3321, which has been submitted to the European Bioinformatics Institute (http://www.ebi.ac.uk/arrayexpress/).
